# Metagenomic identification, isolation, and complete genome characterization of two novel picornaviruses in wild duck from Northeastern Siberia

**DOI:** 10.1186/s12985-025-03017-w

**Published:** 2025-11-22

**Authors:** Nikita Dubovitskiy, Olga Kurskaya, Mariya Solomatina, Arina Loginova, Anastasiya Derko, Anna Khozyainova, Evgeny Denisov, Evgeniy Shemyakin, Alexander Shestopalov, Kirill Sharshov

**Affiliations:** 1 Research Institute of Virology, Federal Research Center of Fundamental and Translational Medicine, Novosibirsk, Russia; 2 Cancer Research Institute, Tomsk National Research Medical Center, Russian Academy of Sciences, Tomsk, Russia; 3 Research Institute of Molecular and Cellular Medicine, RUDN University, Moscow, Russia; 4 Institute of Biological Problems of the Cryolithozone, Siberian Branch, Russian Academy of Sciences, Yakutsk, Russia; 5 Institute of Medicine and Medical Technologies, Novosibirsk State University, Novosibirsk, Russia

**Keywords:** Duck picornavirus, DHAV, Duck hepatitis A virus, Avian virus, Viral metagenomics, Virome, Novel virus

## Abstract

Duck hepatitis A virus (DHAV, *Avihepatovirus ahepati* species) is a well-known pathogen of domestic ducks, causing fatal disease in ducklings and posing a significant burden to duck poultry farms. *Avihepatovirus ahepati* is the sole species within the genus *Avihepatovirus* and is classified into three distinct genotypes: DHAV-1, DHAV-2, and DHAV-3. In our study, we detected and isolated two strains belonging to the *Picornaviridae* family from *Anas crecca* in Northeastern Asia. One of the isolates is related to the DHAV-1 group; however, its polyprotein gene sequence shares only 77.83% nucleotide identity and 89.68% amino acid identity with the most closely related DHAV-1 sequence available, suggesting it represents a highly divergent lineage. The second isolated duck picornavirus shows 60.16% nucleotide identity to the polyprotein gene sequence of the previously described duck picornavirus strain detected during an outbreak in domestic ducks in China. The identification of these two novel picornaviruses in wild ducks, along with their efficient replication in duck embryos and primary cell cultures, emphasizes the need for comprehensive studies of their prevalence in wild ducks and their biological traits to assess potential risks for wildlife and poultry farming. The obtained complete genome sequences and viral isolates enhance our understanding of the diversity, evolution, and ecology of avian picornaviruses.

**Graphical abstract:**

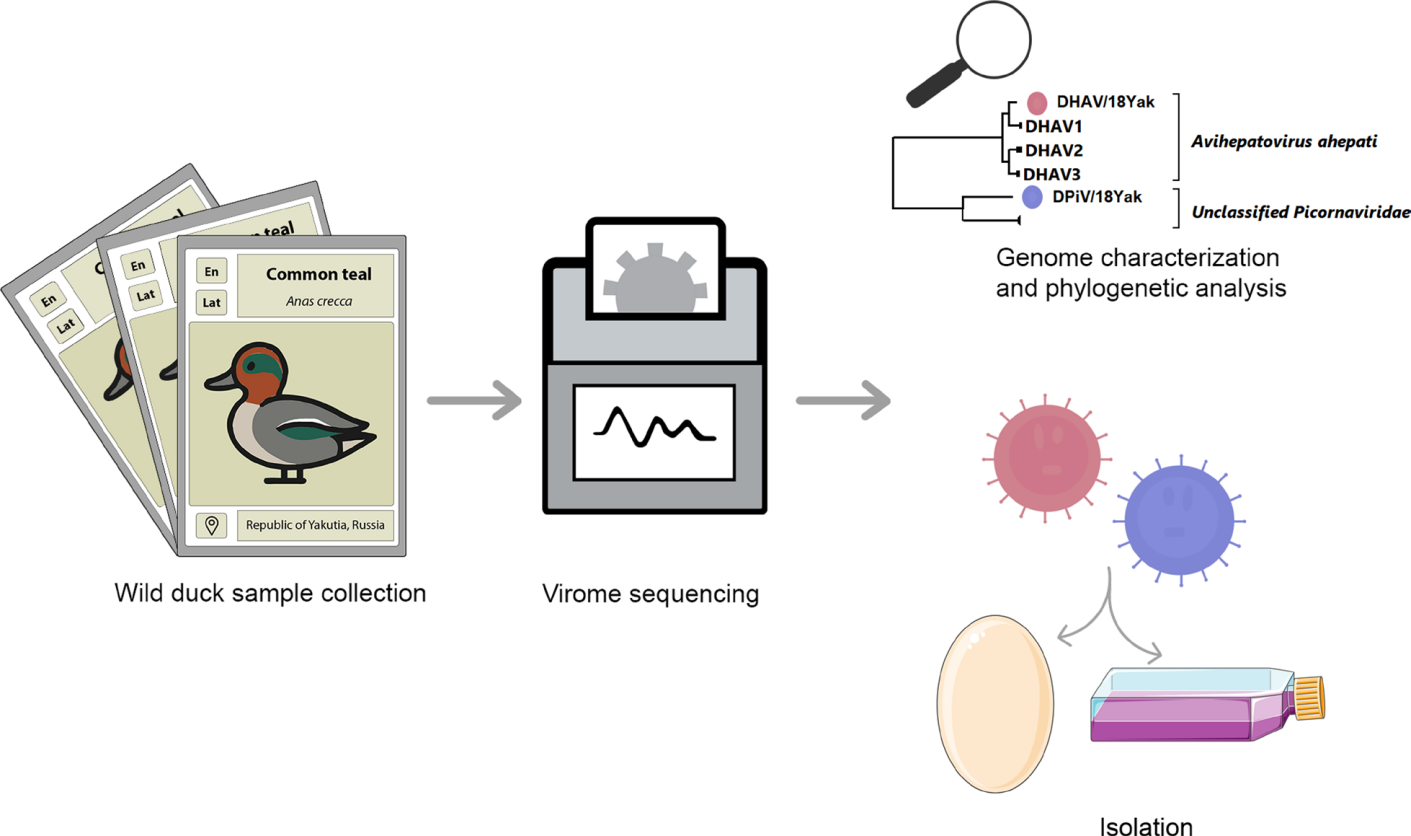

**Supplementary Information:**

The online version contains supplementary material available at 10.1186/s12985-025-03017-w.

## Introduction

Viruses can impose a substantial burden on poultry farming, causing diseases with high morbidity and mortality among birds, and resulting in significant economic losses [1]. A wide variety of avian viruses, including those from the *Astroviridae*, *Orthomyxoviridae*, *Paramyxoviridae*, *Parvoviridae*, and *Sedoreoviridae* families, circulate among wild and domestic bird populations. While some avian viruses have been extensively characterized due to their agricultural and public health importance, many other remain poorly understood, and their diversity, host range, and ecological roles are still being uncovered.

*Picornaviridae* is a family of small non-enveloped viruses with positive-sense single-stranded RNA genome. Following International Committee on Taxonomy of Viruses (ICTV), the *Picornaviridae* family is divided into 159 species belonging to 68 genera based on genomic data. The genome length of *Picornaviridae* species varies from 6.7 to 10.1 kb [2]. The open reading frame (ORF), flanked by 5’ and 3’ untranslated regions (UTRs), encodes a polyprotein that is subsequently processed in structural and non-structural proteins. VPg protein covalently linked to 5’-terminus of genomic RNA.

Viruses of the *Picornaviridae* family are capable of infecting vertebrates, causing diseases in a wide range of animal species as well as in humans [2]. A comprehensive study from China performed an investigation of wild bird cloaca viromes of 3182 birds and found 32 genomes of picornaviruses [3]. At present, four designated picornavirus species have been identified in association with domestic ducks: duck hepatitis A virus (DHAV/*Avihepatovirus ahepati*), duck egg-reducing syndrome virus (DERSV), duck megrivirus (*Megrivirus aturhepa*), duck aalivirus (*Aalivirus apekidu*), duck anativirus (*Anativirus aductai*) [4–7]. The most well-known picornavirus-associated disease in domestic ducks is caused by duck hepatitis A virus. Duck hepatitis A virus is the pathogen that causes Duck (viral) hepatitis type I, while type II and type III are caused by *Astroviridae* family viruses.

The genome length of duck hepatitis A virus is approximately 7.8 kb. The 5’-UTR of DHAV genome contains a type IV internal ribosome entry site (IRES) [8]. The single ORF’s polyprotein product after translation cleaved by viral 2 A protease into 2 subunits: P1 and P2-P3. Next, protease 3 C cleaves: P1 into structural proteins VP0, VP1, and VP3; P2 into non-structural 2A1, 2A2, 2A3, 2B, and 2 C proteins; P3 into non-structural 3 A, 3B, 3 C, and 3D proteins [9]. Genetically, the duck hepatitis A virus is divided into 3 different genotypes DHAV-1, DHAV-2, and DHAV-3. Pathogenetically, DHAV-1 primarily infects 1–2 weeks ducklings causing liver enlargement and hemorrhages. However, another detected pathotype of DHAV-1, designated as pancreatitis-type, induces pancreatitis without pathological changes in liver [10]. Additionally, it has been shown that the DHAV-1 isolate FC16115 caused egg drop and reduced feed consumption in experimentally infected Cherry Valley laying ducks [11]. DHAV-3 was also detected in the spleen and kidney of ducklings, where it exhibited a higher replication level [9]. Notably, a recent study demonstrated the potential vertical transmission of DHAV-1 from infected ducks to their embryos [12].

DHAV-3 has been reported in Korea, Vietnam, and China, whereas DHAV-2 was first identified in Taiwan and subsequently detected in India [9, 13]. DHAV-1 is the most globally widespread genotype detected in Eurasia, Africa, and North America [9].

Recently, with the adoption of a metagenomic approach, several studies have reported the detection of picornaviruses in domestic ducks exhibiting disease symptoms. Picornavirus with a relatively low identity to DHAV was isolated from ducks with short beak and dwarfism syndrome (SBDS) in 2015 in China proposing a new species of *Avihepatovirus* genus [14]. Moreover, another picornavirus was isolated in China in 2017 from domestic ducklings with paralysis and neck twist [15]. The virus was related to the *Avihepatovirus* genus; hence, the amino acid identity was relatively low (polyprotein 41.45%). An Avihepatovirus-like group of viruses was detected in wild ducks of the *Anas* species in Australia, forming a phylogenetically distinct sister clade to known *Avihepatovirus* variants [16].

The territory of Russia is crossed by plural intra- and intercontinental migratory routes of wild waterfowl and serves as breeding areas for birds in the summer season, providing the possibility to transmit and replicate a variety of avian viruses among different bird species. In the Asian territories of Russia, the circulation of avian influenza viruses, avian coronaviruses, avian paramyxoviruses, and avian astroviruses has been documented [17–19]. To date, no genomic sequences of DHAVs circulating in Russia are available in Genbank; however, serological data confirm the presence of DHAV-specific antibodies in duck farms [20].

Therefore, the aim of our study was to perform virological and genetic analysis of newly identified and isolated picornaviruses, including the duck hepatitis A virus, initially detected through metagenomic sequencing of the common teal (*Anas crecca*) feces virome. This study reports the first identification of these viruses in the northern habitats of wild birds (Yakutia region, Russia) and demonstrates their ability to cause mortality in duck embryos. These viruses also exhibit substantial genetic divergence from picornaviruses previously detected in domestic ducks, which are of veterinary significance.

## Materials and methods

### Sample collection and virus-like particles (VLP) enrichment

Feces of 3 common teals (*Anas crecca*) were collected in 2 mL individual tubes in the Yakutia region of Northeast Siberia (Russia) and stored in liquid nitrogen before delivery to the laboratory. Detailed information on the collected samples is provided in Supplementary Material, Table S1. Further preparation was performed following the NetoVir protocol with modifications [21]. A 20% suspension was prepared from individual feces samples. The suspension was centrifuged for 3 min at 17,000 g and 200 µL of supernatant was transferred to a 0.8 μm PES centrifugal filter (Sartorius, Germany). Sample was filtered for 1 min at 17,000 g. Filtrates of 3 samples were pooled in equal volumes and 130 µL of the filtrate was added to a new 1.5 mL tube with 7 µL of 20X nuclease buffer added. Then, 2 µL of bensonase and 1 µL of micrococcal nuclease was added and the sample was incubated for 2 h at 37 °C following termination of treatment with 7 µL of 0.2 M EDTA.

### Detection of viruses via metagenomic approach

Nucleic acid extraction from a VLP-enriched sample was performed with a column-based RNA extraction kit (Biolabmix, Russia) following manufacture protocol. For whole transcriptome amplification, WTA2 kit (Sigma Aldrich, Germany) was used. 2.82 µL of extracted nucleic acids was mixed by pipetting with 0.5 µL of Library Synthesis Solution and incubated at 95 °C for 2 min following cooling to 18 °C. After that 1.68 µL of premixed solution prepared on ice (0.5 µL of Library Synthesis Buffer, 0.78 µL of dH_2_O, and 0.4 µL of Library Solution Enzyme) was added and mixed by pipetting. Sample was incubated at 18 °C for 10 min, 25 °C for 10 min, 37 °C for 30 min, 42 °C for 10 min, 70 °C for 20 min, and 4 °C. Then 7.5 µL of Amplification mix, 60.2 µL of dH_2_O, 1.58 µL of dNTP mix, 0.75 µL of Amplification Enzyme, and 5 µL of DNA library was mixed by pipetting on ice. Samples were incubated in a thermocycler with the following protocol: 94 °C for 2 min, followed by 17 cycles of 94 °C for 30 s, and 70 °C for 5 min, and cooling down to 4 °C. Amplified libraries were purified with a column-based purification kit (Biolabmix, Russia) following manufacture protocol. Further library preparation was performed with a Syntera library preparation kit (Syntol, Russia). Sequencing was performed in Tomsk National Research Medical Center on the Genolab M platform (Genemind, China) and 2 × 150 bp reads were obtained. Paired-end reads were used to obtain metagenomic assembly using ViPER pipeline v.2.3.1 [22] with the following arguments -m 200 –triple-assembly; reads aligned to host genome (*Anas crecca* genome, GCA_036873605) were removed from assembly.

### RT-PCR confirmation of viral RNA presence

We confirmed the RNA presence of viruses detected metagenomically with RT-PCR. For that, we designed oligonucleotides, flanking the region 1093–1455 (DHAVY14F-AGTTTGGTTCCCCTACCCATTCAA; DHAVY14R-ACAGGCTTATGGATTGGTCTCCTC) of detected DHAV genome and the region 2715–3203 (DPIVY14F-GACCAGGGTTTTGATGAGGTGGATT, DPiVY14R-GACATCCCCTTGTGTTAAGCCAGAA) of detected DPiV genome. RNA was isolated with a column-based RNA extraction kit (Biolabmix, Russia) following manufacturer protocol.

BioMaster RT-PCR – Premium kit (Biolabmix, Russia) was used to perform RT-PCR. We added 6.25 µL of RT-PCR-Premium to 0.5 µL of BioMaster-Premium-mix following 5 pmol of each primer, 2.5 µL of RNA, and dH_2_O to a final volume of 12.5 µL. DHAV and DPiV RT-PCRs were performed separately in the temperature profile of reverse transcription at 45 °C for 30 min, preliminary denaturation at 93 °C for 5 min, following 35 cycles: 93 °C for 15 s, 58 °C for 20 s, and 68 °C for 30 s; and final elongation at 68 °C for 7 min.

PCR products were visualized in 1.5% agarose gel electrophoresis.

### Isolation of viruses with duck embryo and duck embryo fibroblast (DEF) primary cell culture

Twelve-day duck embryos were used for virus isolation. A 100 µL volume of the suspension supernatant was inoculated into the allantoic cavity of the embryo. Inoculated embryos were incubated for 5 days at 37 °C. The condition of the embryos was monitored daily by egg candling throughout the incubation period, and allantoic fluid was collected from embryos that died on the day of detection. Collected samples were used to confirm successful isolation and for further assays and passages.

Duck embryo fibroblast (DEF) primary cells from 12-day-old Pekin duck embryos were prepared following protocol for chicken embryo fibroblast isolation as described previously [23] with modifications. Briefly, muscle tissues from duck embryo were cut into 1 mm pieces, washed, and then digested in 0.1% trypsin at room temperature for 10 min, with gentle mixing of the tube every 3 min. Trypsin was removed after centrifugation for 3 min at 5000 g and cells were washed with PBSS 3 times. Prepared DEF cells were seeded into the 96-well plates with 3 × 10^4^ cells per well in a growth medium Dulbecco’s Modified Eagle Medium (DMEM, with 10% FBS (Capricorn Scientific, Germany) and 50 µg/mL of gentamicin sulfate (BioloT, Russia). In 24 h of the incubation cell monolayer was pre-washed with a Hanks solution; then the 100 µL of four-fold dilutions of allantoic fluid containing the virus were inoculated into cells, and plates were incubated at 37 °C and 5% CO_2_ for 1 h for virus adsorption. Upon removing the supernatant, a maintenance medium consisting of DMEM (Capricorn Scientific, Germany) with 2% of FBS (Capricorn Scientific, Germany) and 100 µg/mL of gentamicin sulfate (BioloT, Russia) was added into the wells. Cytopathic effect of viruses on DEFs was assigned visually using the microscope (Micromed I, Russia). At the 4th and 6th d.p.i. aliquots of supernatant were collected for further PCR-detection of the virus.

### ELD50 Estimation for DHAV

We used the method of Reed and Mench to estimate 50% Embryo Lethal Dose (ELD_50_) of detected DHAV. Two-fold serial dilutions of the virus isolate were inoculated into duck embryos, with 100 µL of diluted sample administrated per embryo. The embryos were incubated then for 7 days. ELD_50_ was calculated with the following formula:

$$\:X=(A-50)/(A-B)$$

where A – percent of mortality at the dilution immediately below the target 50% dose.

B - percent of mortality in dilution immediately above the target 50% dose.

### 3’ RACE of DHAV

We used the Mint RACE cDNA amplification set (Evrogen, Russia) to confirm the 3’-end of the DHAV genome. Extracted RNA was used for cDNA synthesis and amplification with Mint RT and Encyclo PCR kits (Evrogen, Russia) following manufacturer protocol. For the 3’-RACE first round, we used DHAVY14SO3-TTAAGTCCCGATGCCCTGTCC oligonucleotide and for the second round, DHAVY14SO4-GAAATTCAACCCTGGGGCGTC was used. For the first round of RACE, we diluted 2 µL of amplified cDNA with 38 µL of dH_2_O. 2 µL of diluted product was added to reaction mix (40 µL of dH_2_O, 5 µL of 10x Encyclo buffer, 1 µL of dNTP mix, 1 µL of 10 pmol/µL DHAVY14SO3, and 1 µL of Encyclo polymerase). We divided the reaction volume into two 200 µL tubes 25 µL each and added 1 µL of 25x Step-out primer mix-1 to one of them. The second tube was used as a control. RACE was performed in the following conditions: 95 °C for 1 min following 29 cycles: 95 °C for 15 s, 58 °C for 20 s, and 72 °C for 3 min. The second step of amplification was performed with a 1:20 diluted amplification product from the first step with DHAVY14SO4 oligonucleotide in the same conditions. The product of amplification was observed with 1.5% agarose gel electrophoresis and confirmed via Sanger sequencing.

### Genome annotation and phylogenetic analysis

The genome sequence of DHAV was annotated with VADR v.1.6.4 and reference DHAV genome NC_008250 [24]. The genome sequence of unclassified DPiV was annotated manually due to low identity using reference DPiV sequence from GenBank (MT681985). Annotated genome sequences were visualized with the DNA Features Viewer tool [25]. Alignment of amino acid sequences of polyprotein was created with MAFFT v.7.505 [26]. The phylogenetic tree was constructed with IQTREE2 v.2.3.6 using a maximum likelihood algorithm and best-fit substitution model (LG + F + I + R4), with node support evaluated through 1,000 bootstrap replicates [27]. The resulting tree was visualized with the ggtree R package [28]. Simplot of complete genome sequences of *Avihepatovirus* was constructed with seqcombo R package. For IRES RNA sequence secondary structure prediction RNAfold of ViennaRNA Package 2.0 [29] was used with default parameters with further manual visualization using RiboSketch [30].

## Results

### Metagenomic detection of picornaviruses presence

Pooled metagenomic sequencing of fecal samples from three common teal individuals generated 19,577,424 paired-end reads. After metagenomic assembly, 764,678 reads were re-aligned to viral contigs, with 138,826 reads assigned to the *Picornaviridae* family. The most abundant contig (Y14_DHAV) belonging to the *Picornaviridae* family was assigned to species *Avihepatovirus ahepati*. The next most abundant contig (Y14_DPiV), supported by 63,006 reads, was identified as Duck picornavirus (DPiV) of an unassigned species. However, the top hit in the BLASTx search corresponded to a Duck picornavirus associated with short beak and dwarfism syndrome in domestic ducklings in China [14]. The length of trimmed contigs was 7731 and 7431 with average coverage of 268× and 235× for Y14_DHAV and Y14_DPiV, respectively. To confirm the presence of viral RNA in the sample and to identify its source within the metagenomic pool, RNA was extracted from individual feces samples comprising the pool. RT-PCR using two pairs of oligonucleotides (DHAVY14, DPiVY14) confirmed the presence of viral RNA corresponding to two detected viruses in a single sample (18Yak).

### Isolation of DHAV and DPiV

Then, we inoculated 12-days duck embryos with feces suspension and after incubation, we confirmed the presence of two viruses in allantoic fluid with RT-PCR. Subsequently, a primary duck embryo fibroblast (DEF) cell culture was used in an attempt to obtain viral monoisolates. A serial 1:4 dilutions of the positive allantoic fluid were inoculated onto DEF cells, but no cytopathic effect (CPE) was observed seven days post-inoculation. However, we performed RT-PCR of individual wells and detected wells with monoisolates of DHAV and DPiV in 1:4, 1:16, and 1:64 dilutions which we named as isolates DHAV/18Yak and DPiV/18Yak, respectively. Monoisolates were used for cultivation in the second passage of DEF. DHAV was detected in the second passage with no CPE, and RT-PCR of DPiV monoisolates was negative in the second passage.

Monoisolates from the first passage of DEF cultivation were inoculated into the allantoic cavity of 12-day duck embryos and after cultivation, both viruses were confirmed to replicate with RT-PCR. Signs of pathogenicity of viruses in duck embryos were observed. The pathogenicity of the DHAV isolate in duck embryos was confirmed by an embryonic lethal dose 50% (ELD_50_) assay, which was determined to be 10^1.55^ ELD_50_/mL. Hence, future characterization of the isolates should include deep sequencing and electron microscopy (EM) to exclude the possibility of co-cultivation with other viruses or bacteria that could influence the observed results.

### Genetic characterization

**Fig. 1:**
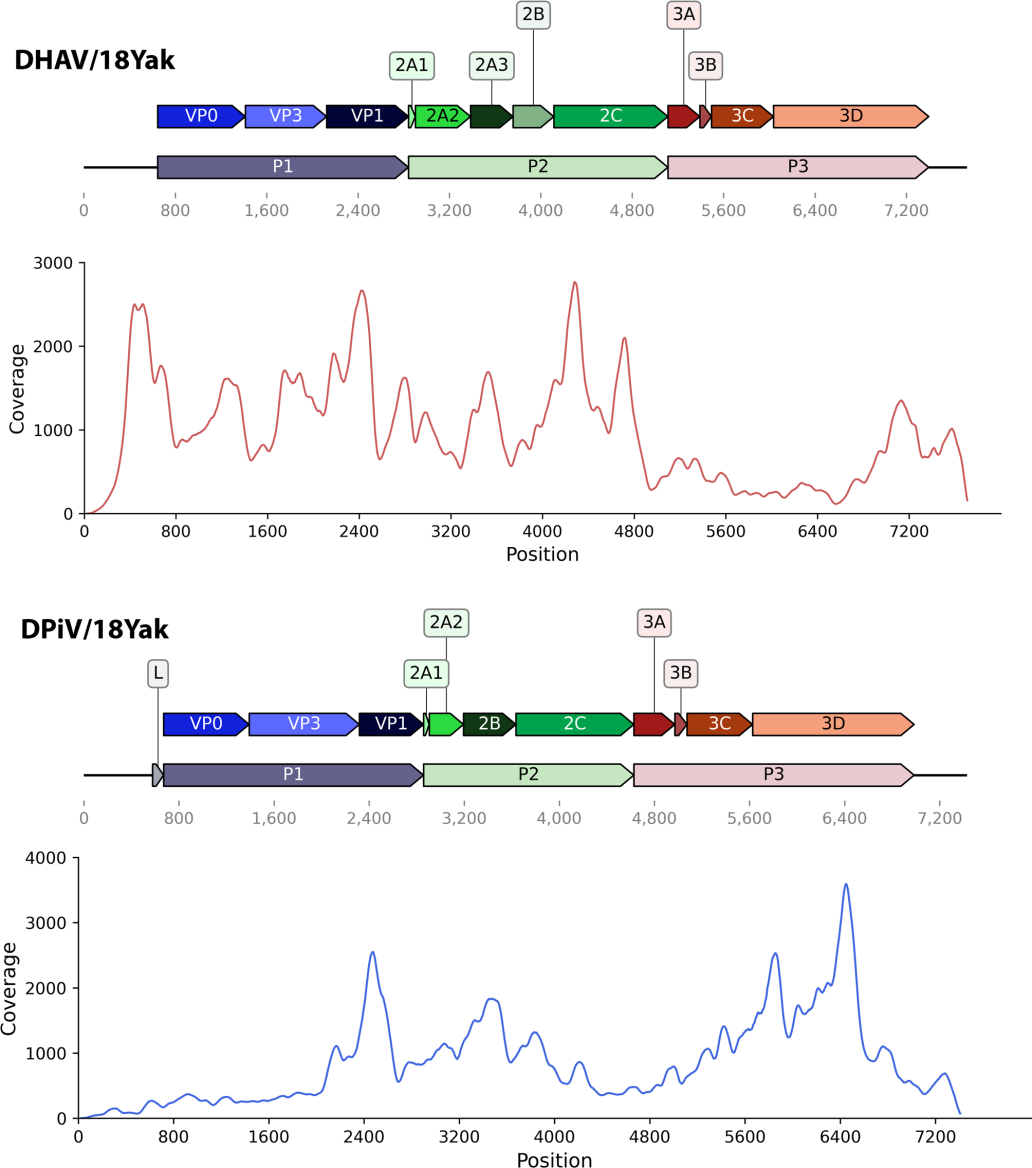
Graphical overview of two picornaviruses genomes obtained in the study – DHAV/18Yak and DPiV/18Yak. Genome coverage by sequencing reads was visualized using a 50-nucleotide sliding window

We confirmed the completeness of the 3’-end of the genome of DHAV with 3’-RACE; however, the 5’-end could not be verified with RACE. The length of contigs and coverage points to the significant reliability of observed genome assembly. DHAV has 5’ UTR of 646 nucleotides and 3’ UTR of 312 nucleotides (polyA excluded). The polyprotein of the detected virus has a length of 6750 nucleotides and 2250 amino acids, respectively, while reference Duck hepatitis A virus type 1 has 2249 amino acids (6747 nucl.). The polyprotein gene identity level is only 77.43% against the reference DHAV-1 sequence (NC_008250), with the maximum value (77.84%) for strain DRL-62 (DQ219396). The polyprotein structure is typical for DHAV: a large polyprotein cleaved onto three subunits P1, P2, P3 – which further cleaved into three structural (VP0, VP3, VP1) and nine non-structural proteins (2A1, 2A2, 2A3, 2B, 2C, 3A, 3B, 3C, and 3D) (Fig. 1).

**Fig. 2:**
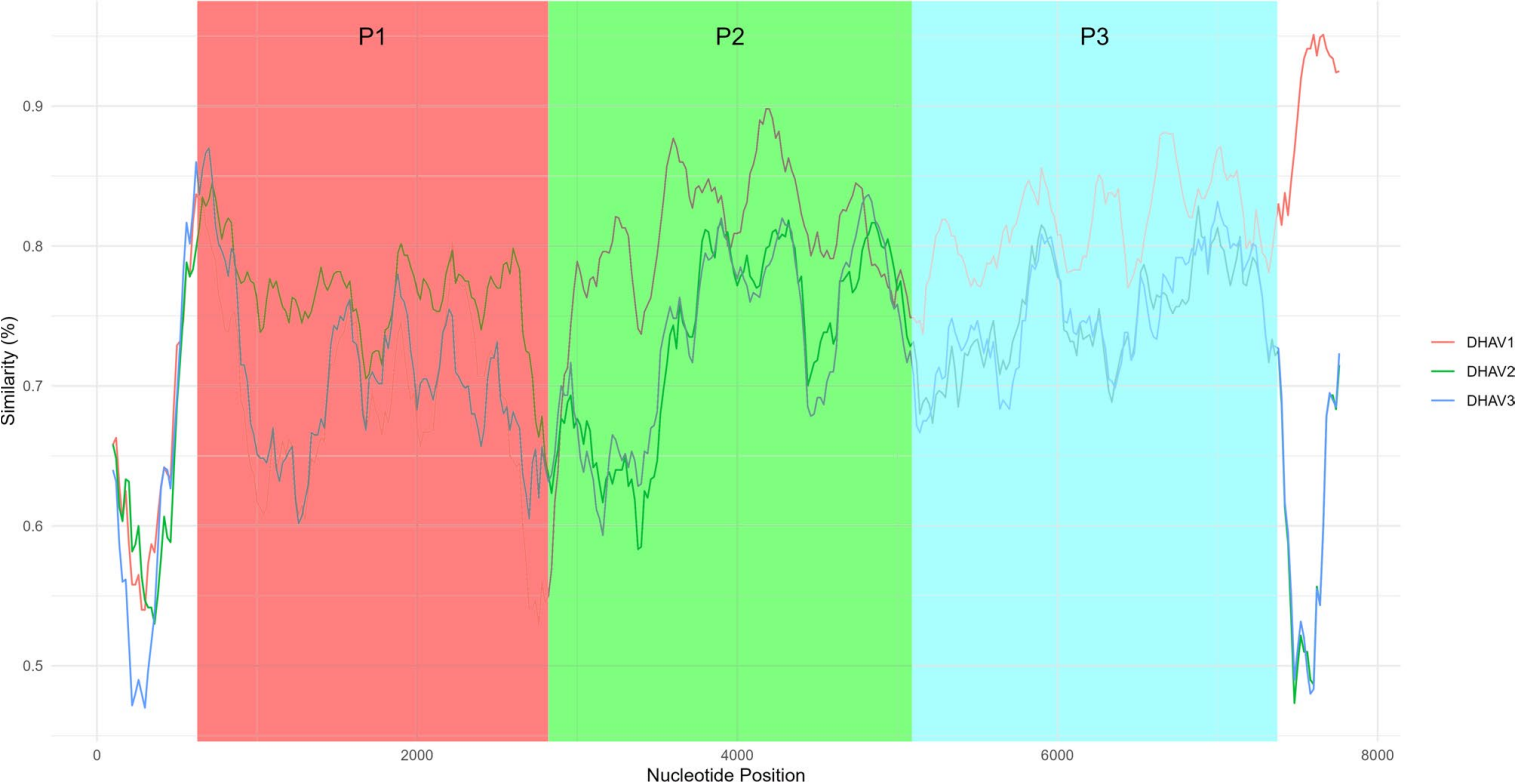
Simplot of complete genome sequences of *Avihepatovirus ahepati* against sequence from the study (DHAV strain 18Yak). Group DHAV1 – DHAV genotype 1 sequences (NC_008250, DQ219396, JF914945, DQ812094, EF585200), group DHAV2 – DHAV genotype 2 (OQ862826, EF067923, EF067924), group DHAV3 – DHAV genotype 3 (DQ256132, JX312194, KU860089)

Interestingly, the P1 region of polyprotein gene encoding structural proteins has the lowest identity values against DHAV-1 reference, while sequences of P1 have higher values of identity in comparison with DHAV-2 (Fig. 2, Figure S2). In particular, the VP1 amino acid sequence of strain from our study has 88.66% of identity with DHAV-2 isolate from India (OQ862826) and only 70.04% of identity with DHAV-1 strain (DQ219396). To investigate potential recombination, six statistical recombination detection methods were applied using RDP5 [31]. Five detection tests supported a recombination event when GENECONV test showed a negative result.

Detailed gene and protein characteristics are provided in Table 1. VP1 protein has the lowest amino acid identity value (70.04%), while 2A3 and 2B are highly conservative with a 100% level of amino acid identity. Previously described conservative region of neutralizing linear B-cell epitope ^75^GEIILT^80^ in DHAV-1 and ^75^GEVILT^80^ in DHAV-3 VP1 differs in detected DHAV − ^77^GELVVT^82^, which has more similarity to DHAV-2 region ^77^GELVIT^82^ [32]. VP3 genotype-1-specific epitope ^205^PSNI^208^ [33] has less similarity with detected strain sequence ^205^PTTI^208^, than similar regions ^205^PNTI^208^ in DHAV-2 and ^205^PSTI^208^ in DHAV-3. Predicted cleavage sites show a 100% level of conservation for 2A2-2A3, 2A3-2B, 2B-2 C, and 3 A-3B regions against DHAV-1 reference. 2A1 protein possesses previously described in DHAV-1 and DHAV-2 strains ribosomal skipping site ^14^GVEPNPGP^21^ [13, 34].

**Table 1:** Detailed genome characteristics of isolated DHAV/18Yak

Gene	Protein	Length, nt/aa	Nt identity	Aa identity	Cleavage site	Reference
Polyprotein	Polyprotein	6750/2250	77.83	89.68		DQ219396
	P1	2196/732	69.60	74.49		
	VP0	768/256	67.85	76.8	*AFND**Q/G**KKKP	
	VP3	711/237	70.60	77.22	*AANN**Q/G**ETNQ	
	VP1	717/239	68.41	70.04	*DLEI**E/T**DQFR	
	P2	2271/757	81.42	97.49		
	2A1	60/20	80.00	90.00	*EPNP**G/P**IMVV	
	2A2	483/161	78.26	93.17	PEFV**S/H**LPRL	
	2A3	372/124	84.95	100	ITTD**Q/S**FPGK	
	2B	357/119	84.31	100	MLED**Q/S**GKTT	
	2 C	999/333	80.68	98.20	*TFMN**Q/S**KVRR	
	P3	2283/761	82.17	96.45		
	3 A	279/93	80.29	97.85	RRFA**Q/S**IYSQ	
	3B	102/34	77.45	91.18	*TELED**Q/S**GRVN	
	3 C	543/181	81.95	97.24	*PVFN**Q/G**KIVS	
	3D	1359/453	83.00	96.25		

*-cleavage site differs from the reference

**Fig. 3:**
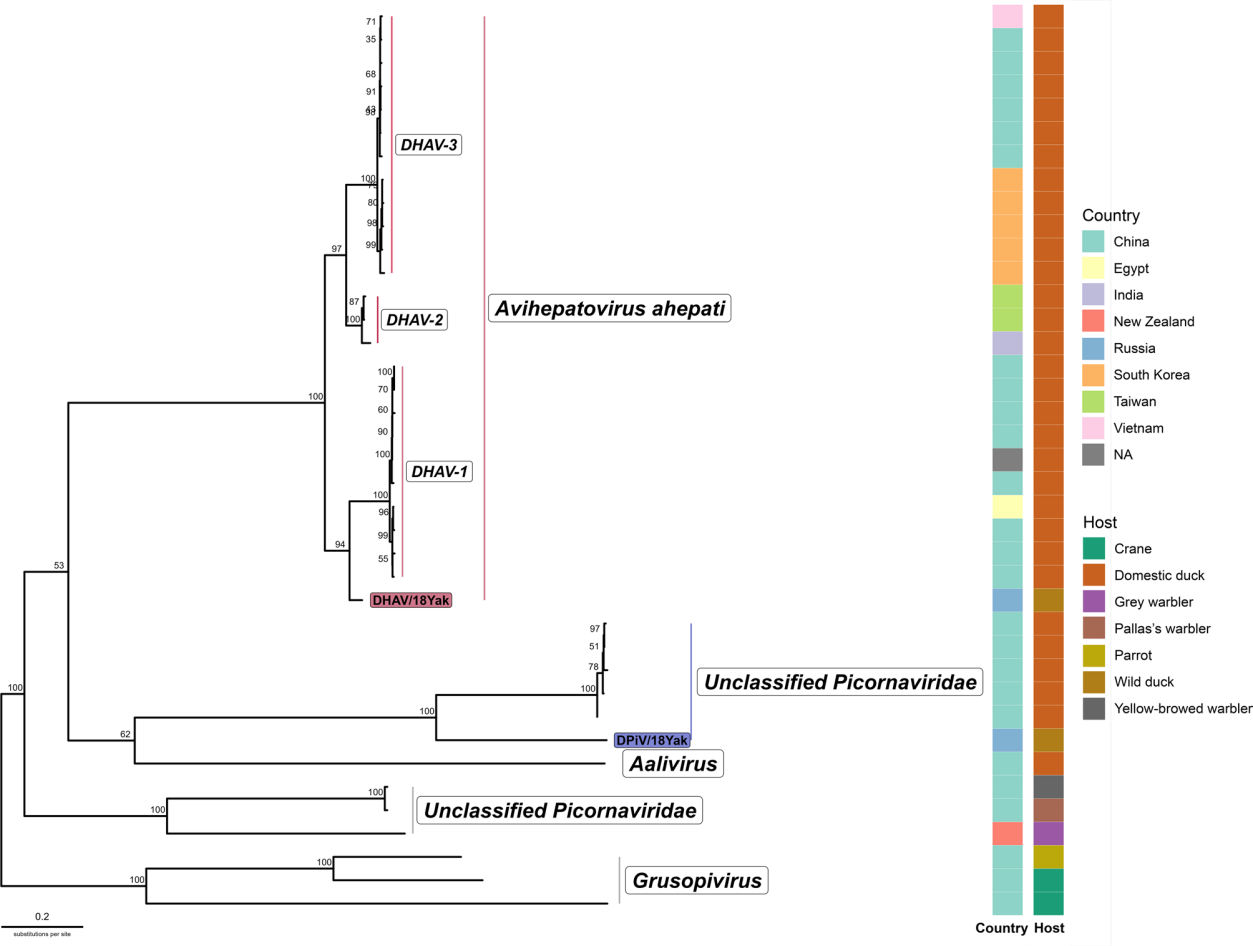
Maximum-likelihood phylogenetic tree of polyprotein amino acid sequences of *Picornaviridae* genera *Avihepatovirus*, *Aalivirus*, *Grusopivirus* and unclassified *Picornviridae*. Viruses, described in the study marked with red and blue rectangles. List of sequences used for phylogenetic analysis provided in Supplementary Material, Table S4

Phylogenetic analysis of DHAV polyprotein amino acid sequences indicates that the newly identified strain shares a most recent common ancestor with the DHAV-1 clade, yet forms a distinct sister branch (Fig. 3). A relatively high distance between the DHAV-1 clade and the detected strain can support the hypothesis of antigenic difference between them, however, the complex antigenic study is needed.

**Fig. 4:**
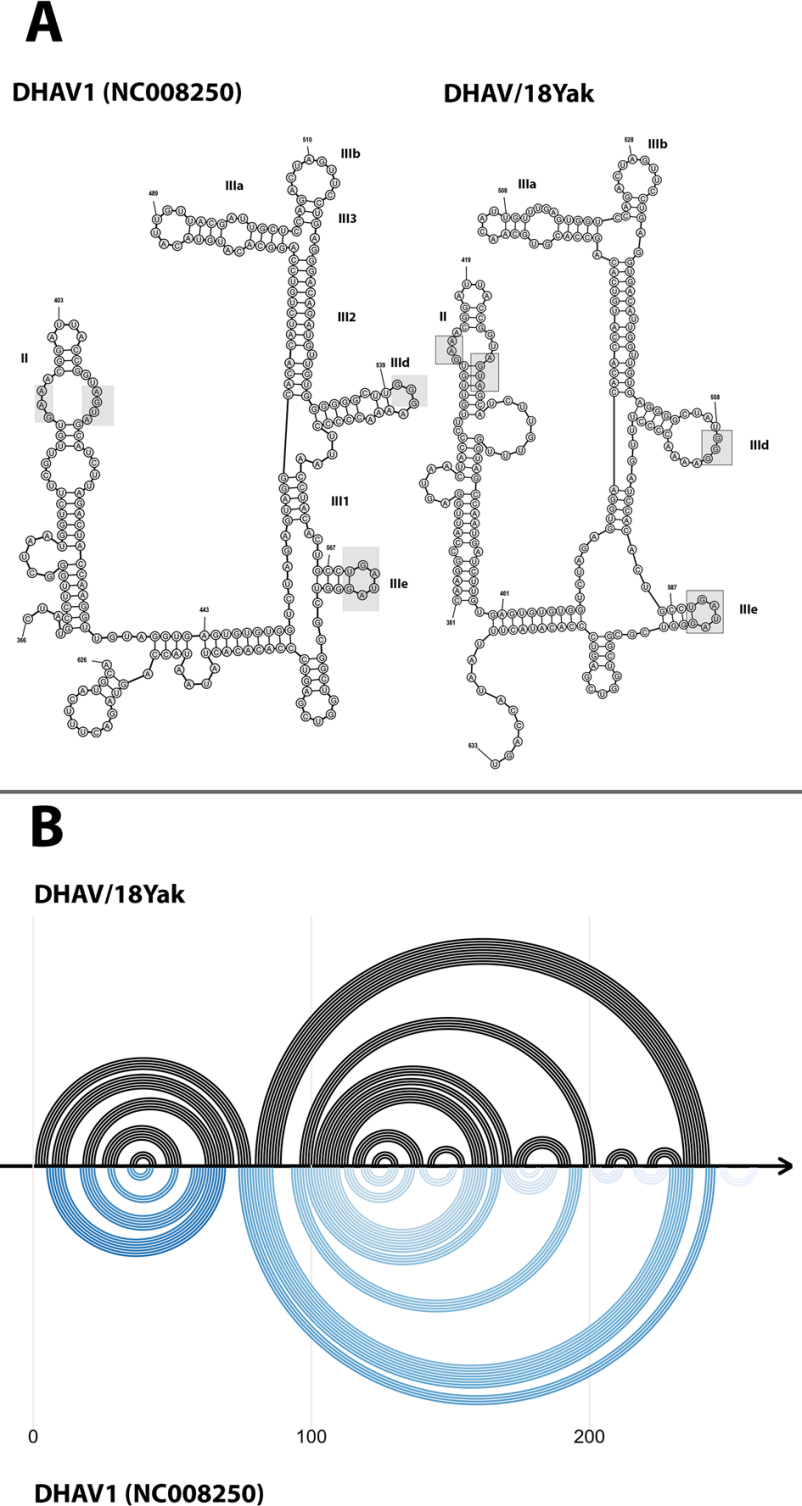
Predicted RNA secondary structure of IRES of DHAV-1 reference strain and DHAV/18Yak strain from the study (**A**). Domains elements are labeled according to [35]; “Loop E” motif in domain II, conserved motifs of domain III are indicated with grey boxes. Pairwise helices comparison plot (**B**)

Similar to previously characterized DHAV strains, the detected DHAV possesses a similar to type IV internal ribosome entry site (IRES) secondary structure in region 361–633 of the genome (Fig. 4). The length of IRES sequence is 266 nucleotides when similar region of reference strain (NC_008250) has 261 nucleotides. Sequences have 83% of nucleotide identity.

The genetic structure of strain DPiV/18Yak is similar to the described Duck/FC22/China/2017 strain: 5’UTR-L-VP0-VP3-VP1-2A1-2A2-2B-2–3 A-3B-3 C-3D-3’UTR. The 5′UTR is 578 nucleotides in length, although its completeness has not been confirmed. The 3′UTR, excluding the poly(A) tail, is 425 nucleotides long. The complete polyprotein of the isolate is 6,405 nucleotides long, encoding 2,135 amino acids, whereas the reference strain contains 6,420 nucleotides and encodes 2,140 amino acids. The strain has a low amino acid identity of polyprotein against the described picornavirus (61.89%) (Table 2). 3 A protein amino acid sequence has the lowest identity level (44.64%) and 2A1 has the highest value (81.25%).

**Table 2:** Detailed genome characteristics of the DPiV/18Yak strain

Gene	Protein	Length, nt/aa	Nt identity, %	Aa identity, %	Reference
Polyprotein	Polyprotein	6405/2135	60.16	61.89	MT681985
	L	93/32	45.16	29.03	
	P1	2187/729	54.41	51.33	
	VP0	720/240	55.82	51.49	
	VP3	927/309	54.75	50.49	
	VP1	540/180	58.67	52.57	
	P2	1770/590	67.61	75.00	
	2A1	48/16	75.00	81.25	
	2A2	288/96	63.54	71.87	
	2B	441/147	67.36	80.69	
	2 C	993/331	67.77	73.11	
	P3	2355/785	61.03	62.87	
	3 A	345/115	54.17	44.64	
	3B	102/34	52.53	54.55	
	3 C	552/184	64.13	69.02	
	3D	1356/452	62.18	65.27	

## Discussion

Identifying virus reservoirs of veterinary importance in the wild poses a significant challenge. For many viruses, virus-host interactions, the role of wild animals in viral evolution, and the diversity of the genetic pool remain poorly understood. Wild migratory birds, particularly those from remote and poorly studied breeding areas in northern latitudes, such as northeast Siberia – serve as important reservoirs. Monitoring these birds is essential due to their potential role in maintaining and transmitting viruses across different ecosystems.

In our study, we detected two avian picornaviruses in common teal feces collected in 2022 in the Northeast region of Russia – Yakutia region. Both viruses were passaged in domestic duck embryos and DEF primary cell culture to obtain two separate monoisolates. The first viral genome was related to the DHAV-1 genotype; however, it exhibited a relatively high pairwise genetic distance from the known DHAV-1 cluster. The coding region of structural proteins (P1 subunit) has 69.60% and 75.68% of nucleotide sequence identity with reference DHAV-1 and DHAV-2 genome, respectively, highlighting the possibility of antigenic differences with known DHAV genotypes, as it codes 3 capsid proteins, where VP1 possesses multiple antigenic epitopes [36]. Particularly, VP1 amino acid sequence similarity of DHAV-1 (DQ219396) in comparison with DHAV-2 (OQ862826) is 70.04%, DHAV-1 with DHAV/18Yak – 70.04%, DHAV-1 with DHAV-3 (DQ812093) – 76.47%. DHAV/18Yak VP1 amino acid sequence shares 88.66% of similarity with DHAV-2. The DHAV/18Yak epitopes in VP1 and VP3 differ from previously described DHAV epitopes, which may additionally indicate potential antigenic divergence. 2A3 and 2B proteins have 100% amino acid identity with the DHAV-1 genome, assuming the conservative role in virus replication. Previous study have reported multiple inter- and intragenic recombination events in DHAVs, with a particularly high frequency detected in the 5’ region and the upstream of the capsid protein-coding region [4]. Simplot, pairwise similarity comparisons, and individual protein phylogenetic trees reveal a relatively close relationship between the P1 region of the isolated DHAV/18Yak and the DHAV-2 genotype (Fig. 2, Supplementary Material: Figure S2, Figure S4), whereas the remainder of the genome shows greater similarity to DHAV-1. Together with the RDP5 recombination tests, this suggests a potential recombination event, however, additional genomic data from the newly identified wild duck-type DHAV cluster is needed to determine whether the observed signal results from recombination or can be attributed to convergent evolution. Understanding the antigenic properties and potential recombination events is crucial for the early detection of novel viral variants that may have significant implications for wildlife diseases and veterinary medicine, particularly as a threat to the health of diverse bird species.

Disease reported for *Avihepatovirus* in China had high mortality and morbidity among 20–25-day-old ducklings with hemorrhage in the liver and pancreas [9]. The virus detected in China was isolated with SPF chicken embryos with 100% mortality in embryos after 4 passages. In our study, we have not observed CPE in DEF inoculated with isolates, however, both isolates showed limited pathogenicity in duck embryos. Despite detected viruses were able to replicate in DEF cells (limited replication for DPiV), we assume that the virus titer was low which corresponds to the data that DHAV can replicate in duck and chicken EF with low efficacy [9]. Low nucleotide identity of viral capsid protein genes against known variants in composition with observed signs of embryonic pathogenicity highlights the possibility of domestic duck infection with wild duck virus variants. However, experimental confirmation using animal model infection is needed.

The second virus genome (DPiV/18Yak strain) was basal to a genomically diverse clade of unclassified picornaviruses of wild birds. The virus with the highest identity level was previously reported to be detected in domestic ducks with short beak and dwarfism syndrome outbreak in China, and based on similarity level, authors propose a new species of *Avihepatovirus* genus [14].

According to the ICTV criteria for the demarcation of picornavirus genera, our DPiV strain qualifies as a new genus based on the protein similarity in P1 being less than 66%, as well as significant differences observed in proteins L, 2B, 3A, and 3B. However, it does not meet the criterion related to similarity levels in proteins 2C, 3C, and 3D, which must be less than 64%.

Considering all the above, we conclude that the DPiV strain belongs to an unclassified genus and represents a distinct species within this genus. This conclusion is supported by: (1) its formation of a sister clade to the unclassified group; and (2) significant protein differences compared to the closest members of the unclassified group.

In summary, based on genome organization and phylogenetic analysis, we propose that one of the isolated viruses belongs to the species *Avihepatovirus ahepati*, sharing its most recent common ancestor with the DHAV-1 genotype. However, it exhibits substantial genetic divergence from known isolates, particularly within the structural protein-coding region, which may reflect antigenic differences. Notably, the virus was isolated from a wild duck, whereas previously characterized strains have been associated with domestic duck populations. Taken together, these findings support the designation of the isolated virus as a novel genotype. Further characterization of the isolate with electron microscopy, metagenomics, and metatranscriptomics deep sequencing to confirm its homogeneity is needed. Additional immunological comparative analysis of the isolate and comprehensive surveillance of DHAV diversity in wild ducks in regions crossed by migratory flyways using new genetic data would provide deeper insights into viral evolution and potential risks. DPiV/18Yak isolate genome has lower identity levels with known viruses and basal even to Unclassified bird picornaviruses clade. Genetic distances partially satisfy ICTV criteria for new genus demarcation in the *Picornaviridae* family. Particularly, P1 protein amino acid sequence identity is less than 64% (51.33%). However, other identity levels are lower than the threshold for genus demarcation. Therefore, although the taxonomy remains uncertain, it is essential to investigate the biological properties of genetically distanced viruses detected in wild ducks in the remote far northeastern region, in order to better assess their potential impact on domestic duck populations.

## Supplementary Information

Below is the link to the electronic supplementary material.

Supplementary Material 1.

## Data Availability

Genome nucleotide sequences of DHAV/18Yak and DPiV/18Yak submitted to GenBank with following accession numbers: PV014360, PV037645.
