# Supplementary material for: Metagenomic identification, isolation, and complete genome characterization of two novel picornaviruses in wild duck from Northeastern Siberia
Source: Virol J. 2025 Nov 22;22:402. doi: 10.1186/s12985-025-03017-w (PMC12751207; doi:10.1186/s12985-025-03017-w)
Supplement: Supplementary file 1 — Supplementary Material 1. [file 12985_2025_3017_MOESM1_ESM.pdf]

Metagenomic Identification, Isolation, and Complete Genome Characterization of Two Novel Picornaviruses in Wild Duck from Northeastern Siberia

Supplementary Material

Table S1. Sample characterization

Pool	Sample	DHAV, PCR	DPiV, PCR	species	date	location
Y14	18	+	+	*Anas crecca*	07.05.2022	Russia: Republic of Yakutia, Oy
	19	-	-	*Anas crecca*	07.05.2022	Russia: Republic of Yakutia, Oy
	20	-	-	*Anas crecca*	07.05.2022	Russia: Republic of Yakutia, Oy

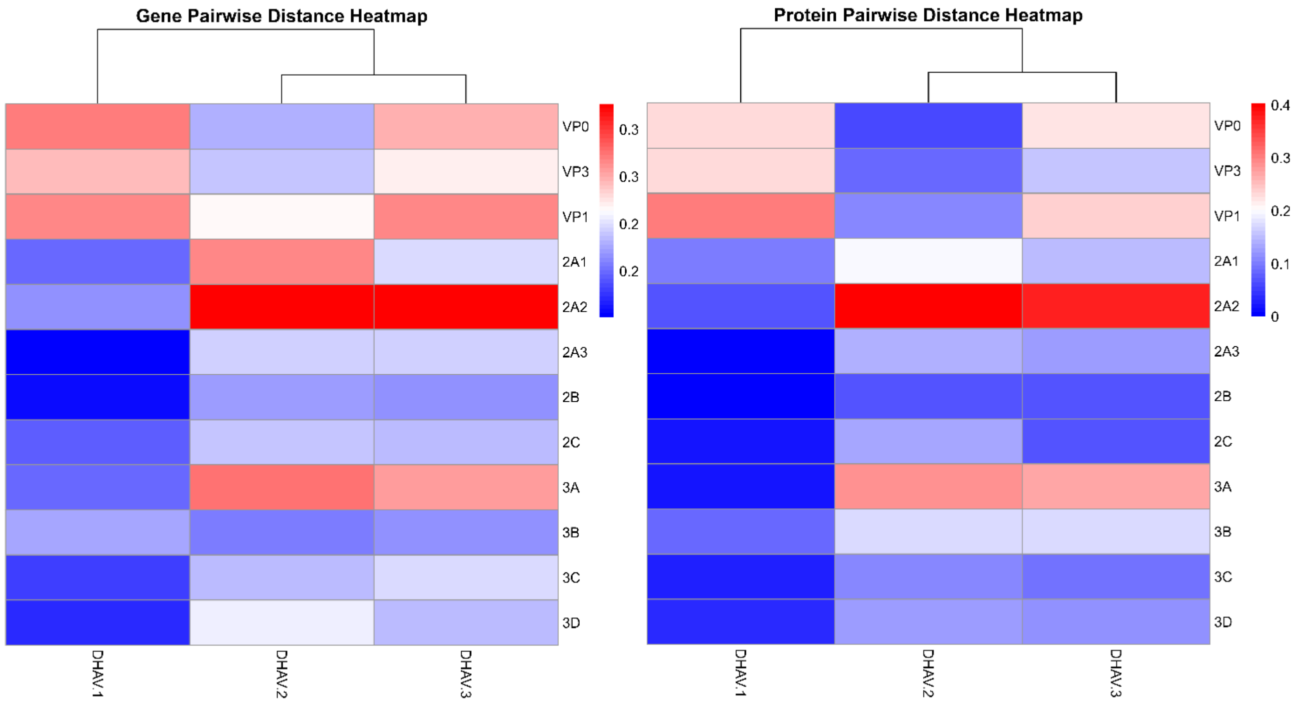

Figure S2. Pairwise distance analysis of the strain DHAV/18Yak from the study against DHAV-1 (DQ219396), DHAV-2 (OQ862826), and DHAV-3 (DQ812093) nucleotide and amino acid sequences.

Table S3. List of sequences used for phylogenetic analysis

Genbank acc.	Virus name	Species
PV014360	DHAV/18Yak	*Avihepatovirus ahepatii*
PV037645	DPiV/18Yak	Unclassified Picornaviridae
MT681985	Duck/AH15/CHN/2015	Unclassified Picornaviridae
OQ927378	Duck/FC16/China/2021	Unclassified Picornaviridae
OQ927379	Duck/FC01/China/2021	Unclassified Picornaviridae
OQ927377	Duck/FC85/China/2021	Unclassified Picornaviridae
MN102111	duck/FC22/China/2017	Unclassified Picornaviridae
KJ000696	GL/12	*Aalivirus apekidu*
NC_075281	Grusopivirus A1 isolate yc-5	*Grusopivirus arecrocra*
NC_075445	Grusopivirus C1 isolate LoPV-1	*Grusopivirus celori*
NC_075282	Grusopivirus B1 isolate yc-6	*Grusopivirus bechini*
MT138358	Picornaviridae sp isolate plw156pic4	Unclassified Picornaviridae
MT138377	Picornaviridae sp isolate ybw202pic01nc	Unclassified Picornaviridae
OQ986628	Avian associated picorna-like virus 25	Unclassified Picornaviridae
EF067924	Duck hepatitis virus 1 strain 90D	*Avihepatovirus ahepatii*
EF067923	Duck hepatitis virus 1 strain 04G	*Avihepatovirus ahepatii*
OQ862826	Avihepatovirus A isolate DHAV/CUL/01/2021	*Avihepatovirus ahepatii*
EU352805	Duck hepatitis A virus 3 strain C-GY	*Avihepatovirus ahepatii*
GU250782	Duck hepatitis virus 1v	*Avihepatovirus ahepatii*
EU877916	Duck hepatitis A virus 3 strain FS	*Avihepatovirus ahepatii*
GQ122332	Duck hepatitis A virus 3 strain GD	*Avihepatovirus ahepatii*
EU755009	Duck hepatitis virus G	*Avihepatovirus ahepatii*
KU860089	Duck hepatitis A virus 3 strain NC	*Avihepatovirus ahepatii*

GQ485311	Duck hepatitis virus SD02	*Avihepatovirus ahepatii*
DQ812093	Duck hepatitis A virus 3 isolate AP-04114	*Avihepatovirus ahepatii*
DQ256132	Duck hepatitis A virus 3 isolate AP-03337	*Avihepatovirus ahepatii*
DQ256133	Duck hepatitis A virus 3 isolate AP-04009	*Avihepatovirus ahepatii*
DQ256134	Duck hepatitis A virus 3 isolate AP-04203	*Avihepatovirus ahepatii*
JX312194	Duck hepatitis A virus 3 isolate D11-JW-018	*Avihepatovirus ahepatii*
NC_008250	Duck hepatitis A virus 1 strain R85952	*Avihepatovirus ahepatii*
MK294345	Avihepatovirus A1 isolate FJ15	*Avihepatovirus ahepatii*
EF151312	Duck hepatitis A virus 1 strain HP-1	*Avihepatovirus ahepatii*
HQ265433	Duck hepatitis A virus isolate SH	*Avihepatovirus ahepatii*
HQ232303	Duck hepatitis virus SH-1/CHN/2010	*Avihepatovirus ahepatii*
EF427900	Duck hepatitis A virus 1 isolate YZ	*Avihepatovirus ahepatii*
EU395440	Duck hepatitis A virus 1	*Avihepatovirus ahepatii*
EU395439	Duck hepatitis A virus 1 isolate ZZ	*Avihepatovirus ahepatii*
EU395438	Duck hepatitis A virus 1 isolate FS	*Avihepatovirus ahepatii*
MZ004919	Avihepatovirus A strain Egypt-14/2019	*Avihepatovirus ahepatii*

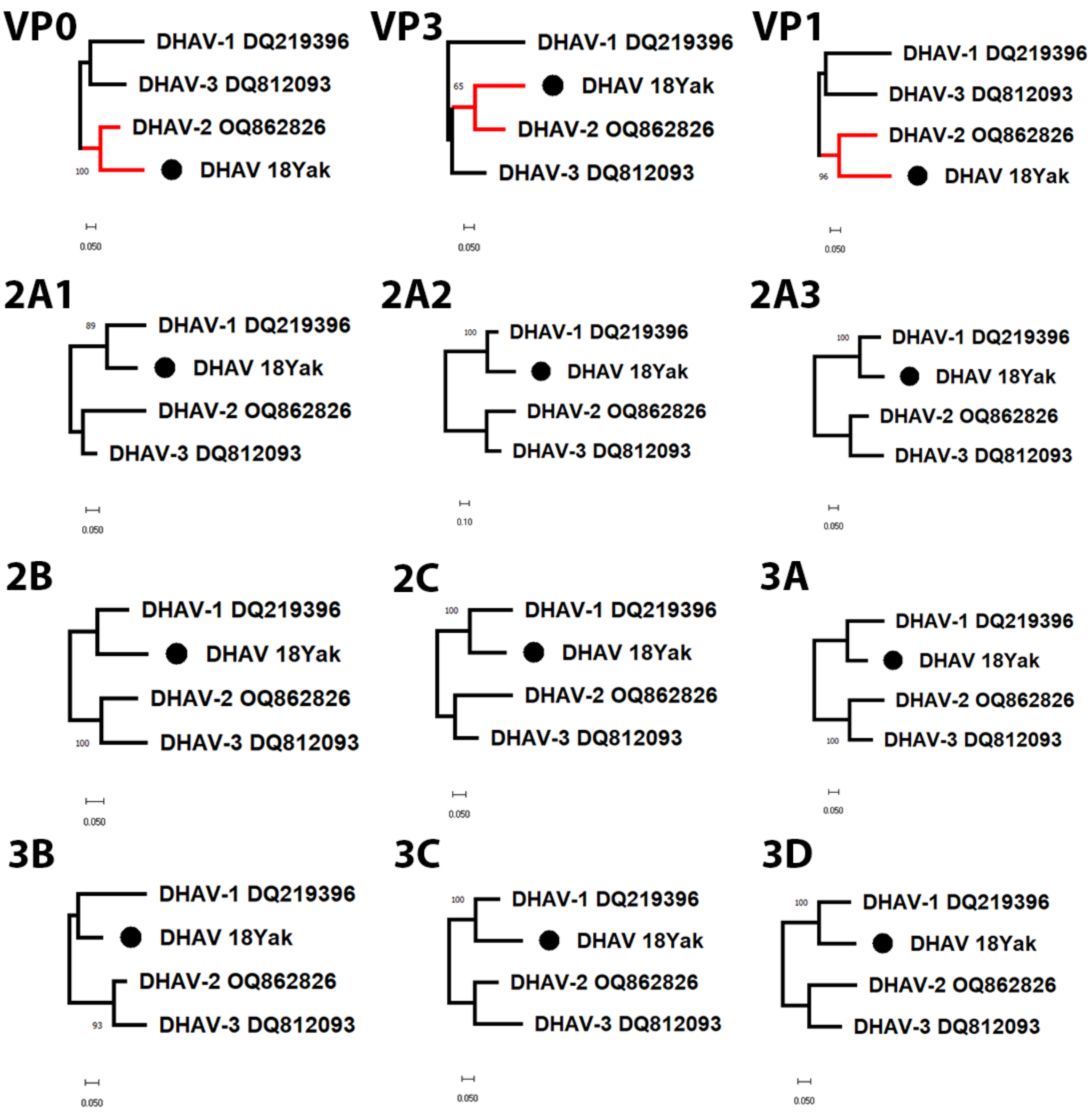

Figure S4. Maximum likelihood trees of nucleotide sequences of the DHAV/18Yak isolate genes with representative DHAV genotypes. DHAV/18Yak marked with black circle. Red branches indicate differences in clustering in the VP0, VP3, and VP1 phylogenetic trees compared with other coding sequences.
